# Mesenchymal stem cell-based therapy and exosomes in COVID-19: current trends and prospects

**DOI:** 10.1186/s13287-021-02542-z

**Published:** 2021-08-21

**Authors:** Mai Abdelgawad, Nourhan Saied Bakry, Ahmed A. Farghali, Ahmed Abdel-Latif, Ahmed Lotfy

**Affiliations:** 1 Biotechnology and Life Sciences Department, Faculty of Postgraduate Studies for Advanced Sciences (PSAS), Beni-Suef University, Beni Suef, 62511 Egypt; 2 Materials Science and Nanotechnology Department, Faculty of Postgraduate Studies for Advanced Sciences (PSAS), Beni-Suef University, Beni Suef, 62511 Egypt; 3 Gill Heart Institute and Division of Cardiovascular Medicine, University of Kentucky and the Lexington VA Medical Center, Lexington, KY USA; 4 College of Medicine, University of Kentucky, Lexington, KY 40506-0046 USA

**Keywords:** Mesenchymal stem cell, Mesenchymal stromal cell, Exosomes, COVID-19, Immunomodulation, Cytokine storm, SARS-CoV-2

## Abstract

Novel coronavirus disease 2019 (COVID-19) is caused by severe acute respiratory syndrome coronavirus-2. The virus causes an exaggerated immune response, resulting in a cytokine storm and acute respiratory distress syndrome, the leading cause of COVID-19-related mortality and morbidity. So far, no therapies have succeeded in circumventing the exacerbated immune response or cytokine storm associated with COVID-19. Mesenchymal stem cells (MSCs), through their immunomodulatory and regenerative activities, mostly mediated by their paracrine effect and extracellular vesicle production, have therapeutic potential in many autoimmune, inflammatory, and degenerative diseases. In this paper, we review clinical studies on the use of MSCs for COVID-19 treatment, including the salutary effects of MSCs on the pathophysiology of COVID-19 and the immunomodulation of the cytokine storm. Ongoing clinical trial designs, cell sources, dose and administration, and populations are summarized, and the paracrine mode of benefit is discussed. We also offer suggestions for optimizing MSC-based therapies, including genetic engineering, strategies for cell surface modification, nanotechnology applications, and combination therapies.

**Supplementary Information:**

The online version contains supplementary material available at 10.1186/s13287-021-02542-z.

## Background

The coronavirus disease 2019 (COVID-19) outbreak emerged in December 2019 in Wuhan, China, but quickly spread worldwide, and the number of cases increased exponentially, with devastating effects on the global economy and public health. The World Health Organization (WHO) designated COVID-19 as a public health crisis because of its high morbidity and mortality (covid19.who.int). According to the Centers for Disease Control and Prevention [1], COVID-19 is characterized by high fever, fatigue, loss of taste and smell, respiratory symptoms, decreased oxygen saturation, and shortness of breath. The causative organism, severe acute respiratory syndrome corona virus-2 (SARS-CoV-2), can also cause neurological disorders, such as encephalopathy, encephalitis, anosmia, ageusia, and Guillain–Barré syndrome, and has been found in the cerebrospinal fluid [2]. COVID-19 can also affect the cardiovascular system, with direct effects on the myocardium and associated myocarditis that causes acute coronary syndrome and myocardial infraction [3]. Some patients suffer from venous thromboembolism and coagulopathy, and these patients in the intensive care unit (ICU) are typically treated with anticoagulation therapy [4–6]. COVID-19 is characterized by cytokine storms, and patients are positive for cytokines, such as monocyte chemoattractant protein 1 (MCP1), macrophage inflammatory protein (MIP)1α, interleukin (IL)-6, IL-2, IL-7, IL-10, and tumor necrosis factor alpha (TNF-α) [7, 8].

There are no approved and effective therapeutics against COVID-19, and scientists are grappling with time to find effective treatments and vaccines. Cell-induced therapies using stem cells, particularly mesenchymal stem cells (MSCs), have been a primary target of therapeutic studies. Many drugs have been repurposed to accelerate drug development [9, 10], while mass vaccination campaigns are being slowly rolled out [11]. Interestingly, the patient response to treatment and therapeutic efficacy has been heterogeneous.

MSCs are self-renewing multipotent stem cells that can differentiate into several cell types. They represent a promising therapy for several chronic lung diseases with high fatality and morbidity rates, such as chronic obstructive pulmonary disease (COPD), obstructive bronchiolitis, idiopathic pulmonary fibrosis, and acute respiratory distress syndrome (ARDS).

Here, we review the use of MSCs as a potential therapy for COVID-19, summarizing their role and immunomodulatory effect in response to a cytokine storm. We discuss completed and ongoing clinical trials and the debate over the use of acellular MSC-based products, such as exosomes, and their effect on COVID-19 pathophysiology. Finally, to improve the chances of treatment success, we suggest methods of enhancing the therapeutic efficacy of MSCs, such as combination therapies, genetic modification and engineering, nanotechnology and nanomaterials, and MSC surface modifications.

### SARS-CoV-2 infection

The novel SARS-CoV-2 [12] shares 79.6% genetic similarity with other human coronaviruses and uses the same target receptor, angiotensin-converting enzyme 2 (ACE 2), for host cell entry [13]. ACE2 is ubiquitously expressed, with high levels in the kidneys, esophagus, colon, small intestine, heart, and lungs [13, 14]. SARS-CoV-2 pathophysiology and virulence are linked to its structural and nonstructural proteins. SARS-CoV-2 can enter type II alveolar cells or other ACE2-expressing cells via the spike (S) protein [15]. Following virus–host cell membrane fusion, viral RNA is released into the host cell, where viral replication, transcription, and translation occur, followed by the assembly of viral proteins and messenger RNAs (mRNAs) into new virions, which are then liberated [12, 16]. Upon SARS-CoV-2 infection, the secreted chemokines induce inflammation of the alveolar and capillary epithelia, causing alveolar and interstitial edema, eventually impairing pulmonary function. Pro-inflammatory granulocytes, monocytes, and macrophages are produced, with reduction in regulatory and anti-inflammatory immune cells. These findings are consistent with histological examinations of lung biopsies that show signs of ARDS, in addition to liver, kidney, and heart damage [13–17].

### COVID-19 and cytokine storms

COVID-19 ARDS is characterized by air exchange dysfunction, edematous changes, secondary infections, and a cytokine storm that can cause multiple-organ dysfunction [13]. There is an increase in leukocytes and inflammatory cytokines and chemokines, such as granulocyte colony-stimulating factor (G-CSF), granulocyte–macrophage colony-stimulating factor (GM-CSF), IL-1β, IL-1 receptor type 1 (IL-1RA), Il-7, IL-8, IL-9, IL-10, fibroblast growth factor 2 (FGF-2), MCP1, vascular endothelial growth factor A (VEGF-A), MIP1-α and MIP1-β, interferon gamma (IFNγ), IFNγ-induced protein 10 (IP10), platelet-derived growth factor B (PDGFB), and TNF-α [18]. Many drugs have been repurposed for COVID-19 treatment but with limited success. As the cytokine storm is the leading cause of death due to COVID-19, immunotherapy seems a favorable treatment option [8]. Tocilizumab (Actemra) is an immunotherapeutic that inhibits IL-6, which plays an integral role in the cytokine storm. However, there is a desperate need for a treatment that can act on a broad range of cytokines [19], and stem cell therapy may be a more beneficial therapeutic approach to treating COVID-19.

### MSCs

In 1966, Friedenstein et al. discovered that fibroblastoids, obtained from murine bone marrow (BM), differentiate into osteocytes when subcutaneously transplanted. Fibroblastoids have since been named MSCs and have regenerative, multilineage differentiation, self-renewal, and immunomodulatory properties in vitro and in vivo, where they form a reservoir of restorative cells. MSCs can migrate to any part of the body, including wound, disease, and inflamed sites, where they modulate an immune response or differentiate into specific cell types [20–24]. MSCs can activate a tissue’s inhabitant stem cells to participate in the healing process [25].

Significant advances have been made in MSC isolation, culture, characterization, and differentiation for exogenous use, due to their low immunogenic profile. MSCs can be isolated from peripheral blood, the umbilical cord, adipose (AD) tissue, and BM [26–29]. They express CD90, CD73, and CD105 but not CD45, CD34, CD14, CD11b, CD79α, and human leukocyte antigen (HLA)-DR [30]. Under specific conditions, MSCs can be expanded in vitro and induced to differentiate into diverse cell types, such as osteoblasts, chondroblasts, ligamentous tissue, neuronal cells, stromal cells, and adipocytes [31].

MSCs have been applied in numerous preclinical and clinical studies [21, 22] to determine their safety and potential for mitigating inflammatory, degenerative, and autoimmune diseases, such as epilepsy, osteoarthritis, multiple sclerosis, rheumatoid arthritis, Crohn’s disease, inflammatory bowel disease (IBD), systemic lupus erythematosus, type 1 diabetes (T1D), autoimmune hepatitis, amyotrophic lateral sclerosis, and corneal epithelial stem cell deficiency [32–44].

### Immunomodulation

MSCs are considered the only stem cell type with immunomodulatory activity and are, therefore, a primary target for therapeutic development for autoimmune disease and inflammation [44]. MSCs secrete immunomodulators, including chemokines, IL-6 and prostaglandin E2 (PGE2), hemoxygenase-1, leukocyte inhibitory factor, indolamine 2,3-dioxygenase (IDO), and transforming growth factor β [45]. MSCs also induce IL-10 expression [46]. Human umbilical cord tissue-derived MSCs (hUC-MSCs) reprogram macrophages and monocytes via cytoplasmic organelles (RNA processing bodies [p-bodies]), a critical lung inflammatory inhibitor. These p-bodies are engulfed by macrophages and monocytes, modulating transcription and inhibiting T cell activation. Low-density lipoprotein receptor-related proteins mediate this interaction on the surface of macrophages and monocytes while blocking pharmacological inhibitors. These findings provide new insight into the inflammatory modulation of MSCs without long-term engulfment by indirectly inhibiting the T cell response through monocyte and macrophage reprogramming by p-bodies [46].

MSCs can migrate to injured and affected tissue. In lung injury, ARDS, and sepsis, MSCs migrate to and are trapped in the lungs, promoting secretion of antimicrobial agents, cytokines, and growth factors [47].

### MSCs and ARDS

**Fig. 1:**
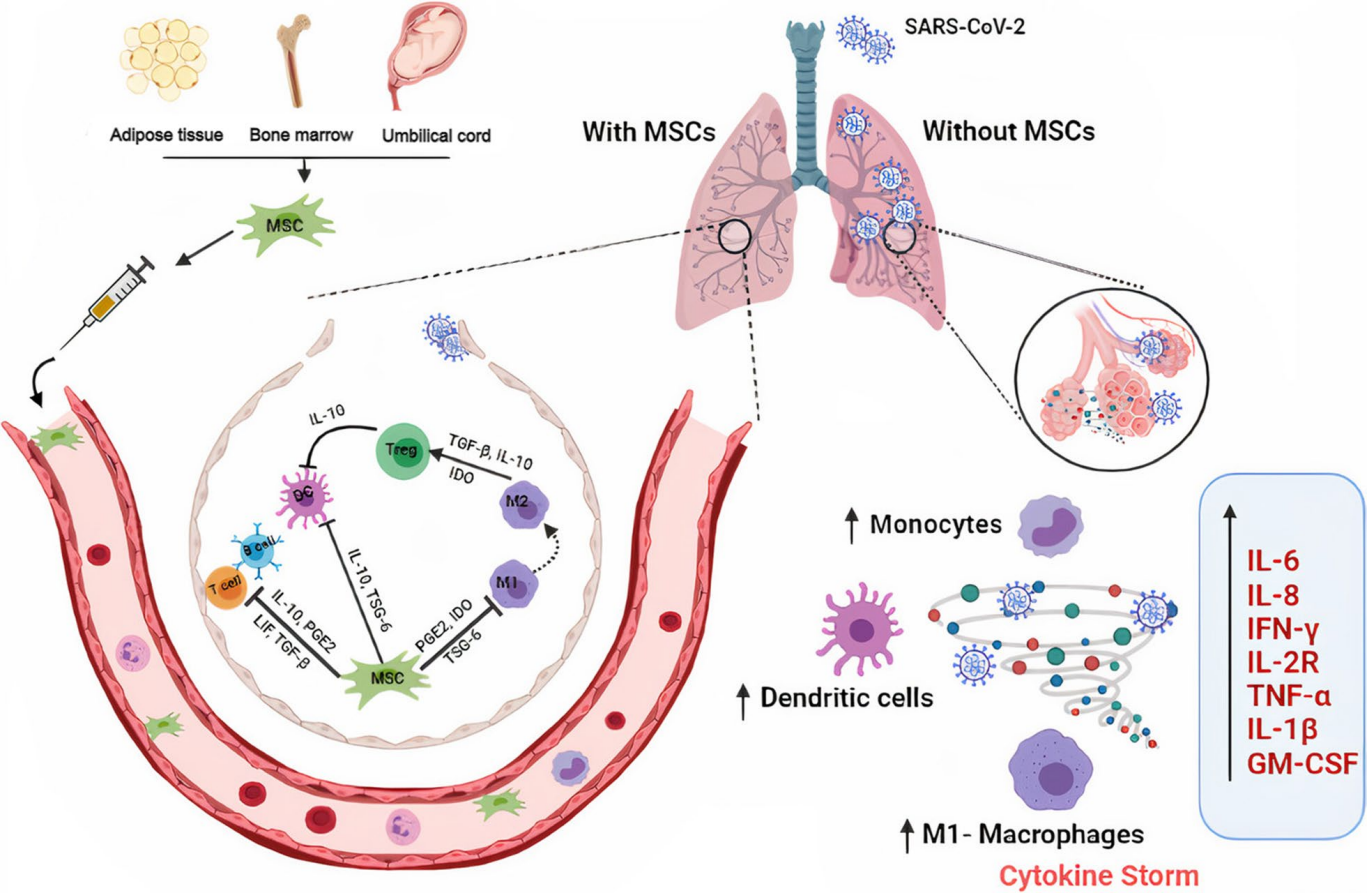
The immunomodulatory role of MSCs in COVID-19. Schematic showing the cytokine storm produced as a consequence of SARS-CoV-2 infection with clarification of the immunomodulatory role of the administrated MSCs in the inflamed lung tissue. The cytokine storm is formed via inflammatory signaling and cytokines and chemokines recruitment. Also, macrophages, dendritic cells and monocytes are activated, leading to severe inflammation, and tissue dysfunction. After MSCs administration, MSCs migrate to the affected tissue and significant secretions of immunomodulatory biomolecules, and cytokines are observed. MSCs can be employed to reduce the produced inflammation via contact-dependent process and paracrine factors’ secretion

Many preclinical and clinical studies have illustrated the therapeutic potential of MSCs in ARDS [48–53]. In a bleomycin-induced lung injury murine model, lung cells were protected from injury and fibrosis by migration of transplanted MSCs to the injury site, where they differentiated into lung cells and inhibited inflammatory cytokine production [48]. In a phase 1 clinical study, the safety of intravenous (IV) infusion of BM-MSCs in moderate-to-severe ARDS patients was validated; however, further studies are required for therapeutic efficacy [52]. MSCs mitigate the cytokine storm via IL-10 and IL-1RA induction and TNF-α and neutrophil influx and assembly inhibition [50, 54]. MSC-secreted keratinocyte growth factor (KGF) induces alveolar epithelial cell repair and proliferation via IL-1RA, GM-CSF, and matrix metalloproteinase-9 (MMP-9) induction [54–56]. VEGF and hepatocyte growth factor (HGF) secreted by MSCs reduce endothelial cell permeation [55, 56]. These findings suggest the potential utility of MSCs as treatment for COVID-19 ARDS patients. The potential beneficial effects are summarized in Fig. 1.

## Studies on MSC therapy for COVID-19

Multiple studies have used MSCs in the COVID-19 setting (Table 1 and Additional file 1). Their therapeutic utility varies by stage of disease:

- Mild: mild clinical manifestations
- Moderate or common: fever, respiratory symptoms, pneumonia on X-ray or computed tomography (CT)
- Severe: respiratory distress (respiratory rate [RR] ≥ 30/min), oxygen saturation ≤ 93% at rest, or arterial partial pressure of oxygen (PaO_2_)/fraction of O_2_ inspiration (FiO_2_) ≤ 300 mmHg
- Critically ill: respiratory failure needing mechanical ventilation, shock, shock with other organ failure, or needing ICU monitoring and treatment

**Table 1:** Published studies of MSC therapy for COVID-19

References	MSCs' source	MSCs' dose and frequency	Study type	Covid-19 stage	Mortality rate in the treatment group versus control group	Respiratory outcomes	General outcomes	Inflammatory markers	Diagnostic testing
[57]	hUMSCs	5 × 10^7^ cells each time–every three days (day 13–16–19)	Case report	*Treatment group*: Critically ill (1) *Control group*: N/A	(0) in (1) versus N/A	The patient was weaned off the ventilator after the second dose of MSCs; denoting an improvement in oxygen saturation	An amelioration regarding the vital signs was reported	A marked decrease in CRP, AST, ALT, d-dimer, WBCs, neutrophils, and bilirubin levels along with an increase in CD3, CD4, and CD8 T cells after MSCs therapy	*X-ray*: showed GGO *PCR*: Positive for COVID-19 *Following MSCs therapy*: *CT*: A relief in the GGO was remarkable by the administration of MSCs
(58)	NR	1 × 10^6^ cells/Kg- Once	Pilot trial	*Treatment group*: Critically ill (1) Severe (4) Common (2) *Control group*: Severe (3)	N/A	Resolution of dyspnea consistent with an increase in oxygen saturation	Resolution of symptoms such as high fever and weakness along with improvement in respiratory rate (in the critically ill patient)	Results (of the critically ill patient) displayed a drop in: CRP, WBCs (including neutrophils along with a rise in lymphocytes) and procalcitonin Bilirubin and AST Creatine kinase and troponin TNF-α Cytokine storm inflammatory cells: CXCR3 + CD4 + T, CXCR3 + CD8 + T, and CXCR3 + NK Analysis of transplanted MSCs showed a rise in: The anti-inflammatory IL-10 The trophic factors: TGF-β, HGF, LIF, GAL, NOA1, FGF, VEGF, EGF, BDNF, and NGF SPA and SPC indicating a differentiation potential	*PCR*: Positive for COVID-19 *CT (for the critically ill patient)*: GGO *Following MSCs therapy:* *CT (for the critically ill patient):* Obvious reduction in the GGO *PCR:* Negative in 4 patients of the treatment group after MSCs therapy
(59)	hUMSCs	2 × 10^6^ cells/kg- once	Pilot trial	*Treatment group:* Severe (12) *Control group:* Severe (29)	*28-day mortality rate:* (0) in (12) versus (3) in (27) N/A	*Only in patients* < *65* A rapid improvement of dyspnea (consistent with an improved oxygen status) in the treatment versus control group	*Only in patients* < *65* A rapid improvement of weakness and fatigue in the treatment versus control group	A significant decline in CRP, IL-6, and a faster improvement of lymphopenia	*CT:* GGO *PCR:* Positive *After therapy:* *CT:* A reduced inflammatory pattern compared to the control group
(61)	UC-MSCs	100 ± 20 × 10^6^ cells 2 doses (day 0 and 3)	A double-blind, phase 1/2a randomized controlled trial	*Treatment group:* Mild-to-moderate (3) moderate-to-severe (9) *Control group:* Mild-to-moderate (3) moderate-to-severe (9)	(2) in (12) versus (7) in (12) by day 28	N/A	Administration of UC-MSCs infusions in COVID-19 with ARDS is safe and accompanied with decreased mortality rate and accelerated recovery time SAEs are improved in UC-MSCs treated group Survival rate at 28 after UC-MSCs treatment was enhanced (91%), while in control (42%)	Dramatic decrease in inflammatory markers (GM-CSF, IFNγ, IL-5, IL-6, IL-7, TNFα-, TNF-β, PDGF-BB, and RANTES) in UC-MSCs treated group with at day 6	*PCR:* a insignificant difference in viral load between treatment groups before and after therapy
(60)	UC-MSCs	3 × 10^7^ cells per dose 3 doses (0, 3, and 6) days	Phase 1 clinical trial	*Treatment group:* Moderate (5), severe (4) *Control group:* Moderate (5), severe (4)	N/A	In UC-MSCs treated group, 1 patient needed mechanical ventilation for 1 day, and another suffered from breath shortness. In control group, 4 patients in mechanical ventilation, and 5 had dyspnea	No SAEs in UC-MSCs treated group UC-MSCs administration in COVID-19 patients was safe and tolerable Enhancement of percentage of inspired oxygen (PaO2/FiO2) ratio in UC-MSCs group	Decrease in inflammatory cytokines (IFN-γ, TNF-α, MCP-1, IP-10, IL-22, IL-1RA, IL-18, IL-8, and MIP-1) in UC-MSCs treated groups within 2 weeks vs control group	*PCR:* Positive *CT or X-ray:* lung lesions showing pneumonia *After therapy:* *PCR:* Negative *SARS-CoV-2 antibody assay (IgM):* Positive in all patients (lower in treatment vs, control group) *CT:* disappearance of lung lesions after 2 weeks of UC-MSCs treatment unlike control
(62)	UC-MSCs	1 × 10^8^ cells 4 doses (one day interval in between)	Pilot trial	*Treatment group:* Severe (9) Critically ill (7)	2 in (16)	Improvement of mean oxygenation index from (258) before MSCs infusion to (329) in day 7 of MSCs infusion	NR	Recovery in lymphocyte counts The measured cytokines (IL-2, IL-4, IL-6, IL-10, TNF-α, IFN-γ and CRP) showed some improvement	*CT or X-Ray*: GGO *PCR:* *SARS-CoV-2 antibody assay:* N/A *After therapy:* *CTor X-ray:* improvement of GGO *PCR:* *SARS-CoV-2 antibody assay:* N/A

Abbreviation: *hUMSCs* human umbilical cord mesenchymal stem cells, *CRP* C-reactive protein, *AST* aspartate aminotransferase, *ALT* alanine transaminase, *GGO* ground-glass opacity, *PCR* polymerase chain reaction, *CT* computed tomography, *NR* not reported, *UC-MSCs* umbilical cord mesenchymal stem cells, *ARDS* acute respiratory distress syndrome, *SAEs* serious adverse events, *ELISA* enzyme linked immunosorbent, *PaO2/FiO2* ratio of arterial oxygen partial pressure to fractional inspired oxygen, *ECG* electrocardiography

One case report described a severely ill patient with COVID-19 who suffered clinical deterioration and was put on a non-invasive mechanical ventilator despite standard therapy. After three doses of 5 × 10^7^ hUC-MSCs on 3 separate days, the patient’s symptoms and laboratory values improved. The patient was weaned off the ventilator 1 day after the second MSC dose. Lymphopenia, including CD3, CD4, and CD8 T cell counts, resolved, with neutrophilia alleviation, and C-reactive protein (CRP), aspartate transaminase (AST), alanine transaminase (ALT), d-dimer, and bilirubin levels decreased [57].

A clinical pilot study of 10 COVID-19 patients (7 in the treatment group and 3 in the control group) evaluated the therapeutic efficacy of IV MSC administration. In the treatment group, one patient was critically ill, four had severe symptoms, and two were moderate cases. After 2–4 days of 10^6^ MSCs/kg administration, all symptoms (fever, fatigue, hypoxia, dyspnea) resolved. CRP levels decreased, oxygen saturation and lymphocytes increased, and cell types that mediate the cytokine storm (CXCR3^+^CD4^+^ T cells, CXCR3^+^CD8^+^ T cells, and CXCR3^+^ natural killer [NK] cells) markedly decreased in the critically ill patient. Regulatory T cells and dendritic cells increased in the critically ill and severely ill patients. No early or delayed adverse events (AEs) were detected. There was a significant drop in pro-inflammatory TNF-α and a significant build-up in IL-10 in the severely ill patients compared with the control group. The proposed mechanism of action was the overexpression of trophic anti-inflammatory factors transforming growth factor beta (TGF-β), HGF, LIF, GAL, NOA1, FGF, VEGF, EGF, BDNF, and NGF. Observed overexpression of SPC and SPA may indicate MSC differentiation to alveolar type II cells [58]. This study suggested the safety and potential efficacy of MSCs as COVID-19 treatment. However, the results were primarily based on the single critically ill patient, so further clinical studies are needed for validation.

In a study on the safety and efficacy of hUC-MSCs as severe COVID-19 treatment, patients were divided into two groups: control (standard treatment; *n* = 29) and treatment (standard treatment + single IV dose of 2 × 10^6^ hUC-MSCs/kg; *n* = 12). The treatment group manifested neither progression from severe to critical disease nor 28-day mortality, whereas four patients in the control group progressed to critical disease and 10.34% of the patients died within 28 days. The treatment group exhibited more rapid improvement in clinical symptoms of dyspnea, weakness, and hypoxemia compared with the control group but only in patients < 65 years old. Laboratory values of CRP, oxygen saturation, IL-6, and lymphocytes (significant between-group differences were detected) and CT further substantiated the therapeutic efficacy of MSCs, and clinical improvement in the treatment group was significant by day 7 [59]. No AEs were reported in the treatment group [59]. However, there were between-group differences in demographics and patient characteristics. In addition, the relatively small sample size might have limited the generalizability of results. Although the time to clinical improvement in the treatment group was significant, the 28-day mortality rate did not differ significantly.

Another study explored the safety and efficacy of hUC-MSCs for moderate and severe COVID-19 treatment in 18 patients: control (standard treatment; *n* = 1) and treatment (standard treatment + 3 × 10^7^ hUC-MSCs/infusion on days 0, 3, and 6; *n* = 9) groups. In the treatment group, mild infusion-related AEs, including fever and flushing, were observed in two patients and transient hypoxia in one patient. Mechanical ventilation was needed in one patient in the treatment group versus four patients in the control group; however, the difference was not significant. No mortality rate was recorded, but inflammatory cytokines reduced. These results prove that IV infusion of MSCs may be safe for moderate and severe COVID-19 treatment [60]. Interestingly, patients with higher IL-6 levels benefited more from hUC-MSC infusion, indicating that a more severe inflammatory environment triggers the MSC immunomodulatory response [60]. MSC activity was believed to occur by reducing inflammatory cytokines; however, a between-group comparison of their levels would have significantly affected the results.

Lanzoni et al. investigated the safety and therapeutic efficacy of hUC-MSCs in 24 COVID-19 patients (12 patients each in the control and treatment groups). In each group, three patients displayed mild-to-moderate ARDS, and nine displayed moderate-to-severe ARDS. In the treatment group, patients were given two IV infusions of 100 ± 20 × 10^6^ hUC‐MSCs at days 0 and 3 plus standard treatment. No treatment-related AEs in terms of infusion-related reactions within the first 6 h, cardiac arrest or death within 24 h, or any other AEs were documented. There was a significant reduction in patient mortality, event-free survival, and time to recovery in the treatment group compared with the control group. A significant drop in the level of inflammatory cytokines and growth factors between days 0 and 6 further confirmed and justified the results. Therefore, hUC-MSCs are safe and effective in COVID-19 treatment [61].

A pilot, single-arm trial in 16 patients with severe and critically severe COVID-19 was performed. After four rounds of hUC-MSC transplantation, patients showed increased oxygen saturation, no allergic reactions, and cytokine storm improvement, demonstrating the safety and feasibility of hUC-MSCs in severe COVID-19 treatment [62].

In summary, monitoring of COVID-19 severity and recovery after MSC administration (Table 1) indicated that MSCs can ameliorate COVID-19 severity and patients can be weaned off the ventilator.

## Ongoing clinical trials of MSC therapy for COVID-19

There are more than 55 ongoing clinical trials to assess the therapeutic efficacy and safety of MSCs in COVID-19 (clinicaltrial.gov; Table 2 and Additional file 2) [63]. The majority of the trials are between phases 1 and 2, and few are in phase 3 [63]. The primary focus is severe COVID-19 because of high mortality and the emergence of promising treatments to decrease disease severity and mortality rates; moderate stage cases are also under study [63]. Most include male and female patients aged 18 years and older, primarily between 50 and 80 years old, while some include children and teenagers as well, and a few are limited to a specific age range [63]. Some of the trials have a low sample size (5–10 patients), while others have low-to-moderate (16–50 patients) or moderate (50–100 patients) sample sizes, which may not reflect the true impact of MSC therapy. However, trials using a large sample size (100–400 patients) may clearly reflect the effect of MSC therapy [63].

**Table 2:** The ongoing clinical trials with MSCs interventions in COVID-19 patients

Clinical trial ID	Status	Phase	Disease Stage	Study type (Interventional/ observational/expanded access)	Stem Cell Type	Source (Allogenic- Autologous)	Route of Administration	Number of doses	Cells/DOSE	Enrollment /sample Size	Country	Allocation	Controlled or Uncontrolled
NCT04461925	Recruiting	Phase 1 Phase 2	Severe	Interventional	hU-MSCs	Allogenic	IV	3	1.0*10e6/kg	30	Ukraine	Non-randomized	Controlled
NCT04452097	Not yet recruiting	Phase 1 Phase 2	Severe	Interventional	hU-MSCs (BX-U001)	Allogenic	IV	1 (either one from the 3)	0.5*10e6, 1.0*10e6, or 1.5 × 10e6 cells/kg	39	N/A	Non-randomized	Uncontrolled
NCT04377334	Not yet recruiting	Phase 2		Interventional	BM-MSCs	Allogenic		N/A	N/A	40	Germany	Randomized	Controlled
NCT04331613	Recruiting	Phase 1 Phase 2	Severe	Interventional	hESCs-derived-IMRCs (CAstem)	Allogenic	IV	1–3 Doses	3*10e6, 5*10e6, or 10*10e6 cells/kg	9	China	N/A	Uncontrolled
NCT04390139	Recruiting	Phase 1 Phase 2	Moderate	Interventional	WJ-MSCs	N/A	Endovenous	2	1*10e6 cells/kg	30	Spain	Randomized	Controlled
NCT04371393	Recruiting	Phase 3	Moderate to severe	Interventional	BM-MSCs (Remestemcel-L)	Allogenic	IV	2	2*10e6 cells/kg	300	United States	Randomized	Controlled
NCT04348461	Not yet recruiting	Phase 2	Severe (on Mechanical ventilation)	Interventional	AT-MSCs	Allogenic	IV	2	1.5*10e6 cells/kg	100	Ukraine	Randomized	Controlled
NCT04400032	Recruiting	Phase 1	Severe (on Mechanical ventilation ≤ 48 h)	Interventional	BM-MSCs	N/A	IV	3	25*10e6, 50*10e6, or 90*10e6 cells	9	Canada	Non-randomized	Uncontrolled
NCT04398303	Not yet recruiting	Phase 1 Phase 2	Moderate to severe	Interventional	hU-MSCs (ACT-20), ACT-20-CM or hU-MSCs in CM (ACT-20)	Allogenic	IV	N/A	1*10e6 cells/kg of hU-MSCs (ACT-20) in 100 ml CM, 100 ml CM of ACT-20-CM	70	N/A	Randomized	Controlled
NCT04393415	Recruiting	Not applicable	Any	Interventional	hU-MSCs	N/A	N/A	N/A	N/A	100	Egypt	Randomized	Controlled
NCT04447833	Recruiting	Phase 1	Severe	Interventional	BM-MSCs (KI-MSC-PL-205)	Allogenic	IV	1	1*10e6 or 2*10e6 cells/kg	9	Sweden	N/A	Uncontrolled
NCT04397796	Recruiting	Phase 1	Moderate to severe	Interventional	BM-MSCs	Allogenic	N/A	N/A	N/A	45	United States	Randomized	Controlled
NCT04467047	Not yet recruiting	Phase 1	Severe	Interventional	BM-MSCs	Allogenic	IV	N/A	1*10e6 cells/kg	10	N/A	N/A	Uncontrolled
NCT03042143	Recruiting	Phase 1 Phase 2	Moderate to severe	Interventional	hU-MSCs (Orbcel-C)	Allogenic	IV	N/A	400*10e6 cells	75	United Kingdom	Randomized	Controlled
NCT04345601	Not yet recruiting	Early Phase 1	Moderate to severe	Interventional	MSCs	Allogenic	IV	1	2*10e6 cells/kg	30	United States	Randomized	Controlled
NCT04269525	Recruiting	Phase 2	Severe or critical	Interventional	hU-MSCs	N/A	IV	4	9.9*10e7 cells	16	China	N/A	Uncontrolled
NCT04365101	Recruiting	Phase 1 Phase 2	Moderate	Interventional	hP-MSCs (CYNK-001)	Allogenic	IV	3	N/A	86	United States	Randomized	Controlled
NCT04361942	Recruiting	Phase 2	Severe	Interventional	MSCs	Allogenic	IV	1	10e6 cells/kg	24	Spain	Randomized	Controlled
NCT04389450	Recruiting	Phase2	Severe	Interventional	hP-MSCs (PLX-PAD)	Allogenic	IM	1 or 2	N/A	140	United States	Randomized	Controlled
NCT04333368	Active, not recruiting	Phase 1 Phase 2	Severe	Interventional	WJ-MSCs		IV (either centrally or peripherally)	3	10e6 cells/kg	47	France	Randomized	Controlled
NCT04367077	Recruiting	Phase 2 Phase 3	Moderate to severe	Interventional	BM-MSCs (MultiStem)	Allogenic	IV	N/A	N/A	400	United States	Randomized	Controlled
NCT04445220	Recruiting	Phase 1 Phase 2	Any	Interventional	Extracorporeal MSCs (SBI-101)	Allogenic	IV	1	250*10e6 or 750*10e6 cells	22	N/A	Randomized	Controlled
NCT04466098	Recruiting	Phase 2	Moderate to severe	Interventional	MSCs	N/A	IV	3	300*10e6 cells	30	United States	Randomized	Controlled
NCT04276987	Completed	Phase 1	Severe	Interventional	AT-MSCs -Exo	Allogenic	Inhalation	5	2*10e8 nanovesicles/3 ml	24	China	N/A	Uncontrolled
NCT04313322	Recruiting	Phase 1		Interventional	WJ-MSCs	Allogenic	IV	3	1*10e6 cells/kg	5	Jordan	N/A	Uncontrolled
NCT04473170	Completed	Phase 1 and Phase 2		Interventional	Non-Hematopoietic Peripheral Blood Stem Cells (NHPBSC)	Autologous	jet nebulization	N/A	N/A	146	United Arab Emirates	Randomized	Controlled
NCT04428801	Not yet recruiting	Phase 2		Interventional	AT-MSCs	Autologous	IV	3	200*10e6 cells	200	N/A	Randomized	Controlled
NCT04486001	Not yet recruiting	Phase 1	Severe	Interventional	AT-MSCs	Allogenic	IV			20	United States	N/A	Uncontrolled
NCT04444271	Recruiting	Phase 2	Moderate	Interventional	MSCs	N/A	IV	1 or 2	2*10e6 cells/kg	20	Pakistan	Randomized	Controlled
NCT04416139	Recruiting	Phase 2	Severe	Interventional	MSCs	Allogenic	IV	1	1*10e6 cells/kg	10	Mexico	Non-randomized	Controlled
NCT04336254	Recruiting	Phase 1 Phase 2	Severe	Interventional	DPSCs	Allogenic	IV	3	3.0*10e7 cells	20	China	Randomized	Controlled
NCT04429763	Not yet recruiting	Phase 2	Severe	Interventional	hU-MSCs	N/A	N/A	1	1*10e6 cells/Kg	30	United States	Randomized	Controlled
NCT04315987	Not yet recruiting	Phase 2	Severe	Interventional	MSCs (NestaCell®)	allogenic	IV	4	2*10e7 cells	90	Brazil	Randomized	Controlled
NCT04456361	Active, not recruiting	Early Phase 1	Mild–moderate–severe	Interventional	WJ-MSCs	N/A	IV	1	1*10e8 cells	9	Mexico	N/A	Uncontrolled
NCT04349631	Active, not recruiting	Phase 2	N/A	Interventional	AT-MSCs	Autologous	IV	5	N/A	56	United States	N/A	Uncontrolled
NCT04366323	Active, not recruiting	Phase 1 Phase 2	severe	Interventional	AT-MSCs	Allogeneic	IV	2	80*10e6 cells	26	Spain	Randomized	Controlled
NCT04348435	Enrolling by invitation	Phase 2		Interventional	AT-MSCs (Hope Biosciences-MSCs)	Allogeneic	IV	5	200*10e6 cells	100	United States	Randomized	Controlled
NCT04252118	Recruiting	Phase 1		Interventional	MSCs	N/A	IV	3	3*10e7 cells	20	China	Non-RANDOMIZED	Controlled
NCT04273646	Not yet recruiting	Not applicable	Severe	Interventional	hU-MSCs	N/A	IV	4	0.5*10e6 cells/kg	48	China	Randomized	Controlled
NCT04382547	Enrolling by invitation	Phase 1 Phase 2	Severe	Interventional	OM-MSCs	Allogeneic	IV	N/A	N/A	40	Belarus	Non-randomized	Controlled
NCT04346368	Not yet recruiting	Phase 1 Phase 2	Severe	Interventional	BM-MSCs	N/A	IV	1	1*10e6 cells/kg	20	China	Randomized	Controlled
NCT04288102	Completed	Phase 2	Severe	Interventional	hU-MSCs		IV	3	4.0*10e7 cells	100	China	Randomized	Controlled
NCT04527224	Not yet recruiting	Phase 1 Phase 2	Moderate	Interventional	AT-MSCs (AstroStem-V)	Allogenic	N/A	N/A	N/A	10		N/A	Uncontrolled
NCT04339660	Recruiting	Phase 1 Phase 2		Interventional	hU-MSCs	N/A	IV	1	1*10e6 cells/kg	30	China	Randomized	Controlled
NCT04457609	Recruiting	Phase 1	Severe	Interventional	hU-MSCs	N/A	IV	1	1*10e6 cells/kg	40	Indonesia	Randomized	Controlled
NCT04366063	Recruiting	Phase 2 Phase 3	Mild to moderate	Interventional	MSCs	N/A	IV	2	100*10e6 (± 10%) cells	60	Islamic Republic of Iran	Randomized	Controlled
NCT04490486	Not yet recruiting	Phase 1		Interventional	hU-MSCs	N/A	IV	2	100*10e6 cells	21	United States	Randomized	Controlled
NCT04355728	Completed	Phase 1 Phase 2	Severe	Interventional	hU-MSCs		IV	2	100*10e6 cells	24	United States	Randomized	Controlled
NCT04535856	Recruiting	Phase 1	Mild or moderate	Interventional	DW-MSC	allogeneic	IV	2	Low-dose group (5*10e7 cells) High-dose group (1*10e8 cells)	9	Indonesia	Randomized	Controlled
NCT04537351	Recruiting	Phase 1 Phase 2		Interventional	iPSCs-derived-MSCs (Cymerus MSCs)	Allogenic	IV	2	2–200*10e6 cells/kg	24	Australia	Randomized	Controlled
NCT04524962	Recruiting	Phase 1 Phase 2	Moderate-to-severe	Interventional	RNA-engineered MSCs to secrete DNases		N/A	N/A	N/A	30	United States	N/A	Uncontrolled
NCT04371601	Active, not recruiting	Early Phase 1	Severe	Interventional	hU- MSCs		IV	4	10e6 cells/Kg	60	China	Randomized	Controlled
NCT04522986	Not yet recruiting	Phase 1	Severe	Interventional	MSCs		IV	4	1*10e8 cells	6	Japan	N/A	Uncontrolled
NCT04362189	Active, not recruiting	Phase 2		Interventional	AT-MSCs (Hope Biosciences-MSCs)	Allogeneic	IV	4	100*10e6 cells	100	United States	Randomized	Controlled
NCT04390152	Not yet recruiting	Phase 1 Phase 2	Moderate to severe	Interventional	WJ-MSCs	N/A	IV	2	50*10e6 cells	40	Colombia	Randomized	Controlled
NCT04611256	Recruiting	Phase 1	Moderate to severe	Interventional	AT-MSCs	N/A	IV	2	1*10e6 cells/kg	20	Mexico	Randomized	Controlled
NCT04565665	Recruiting	Phase 1	Moderate to severe	Interventional	hU-MSCs		IV	1 or 2	N/A	70	United States	Randomized	Controlled
NCT04573270	Completed	Phase 1	N/A	Interventional	MSCs	N/A	IV	1	N/A	40	United States	Randomized	Controlled
NCT04629105	Recruiting	Phase 1	Mild to severe	Interventional	BM-MSCs (Longeveron MSCs)		IV	3	100*10e6 cells	70	United States	Randomized	Controlled
NCT04302519	Not yet recruiting	Early Phase 1	Severe	Interventional	DP-MSCs	N/A	IV	3	1.0*10e6 cells/kg	24	China	Randomized	Uncontrolled
NCT04494386	Recruiting	Phase 1 Phase 2	Mild–moderate–severe	Interventional	hU-MSCs	Allogeneic	IV	1 or 2	100*10e6 cells	60	United States	Randomized	Controlled
NCT04392778	Recruiting	Phase 1 Phase 3	Severe	Interventional	MSCs	N/A	IV	3	3*10e6 cells/kg	30	Turkey	Randomized	Controlled
NCT04657458	Available		Severe	Expanded access	BM-MSC -Derived-ECV(ExoFlo™)	N/A	IV	N/A	N/A	N/A		N/A	N/A
NCT04299152	Not yet recruiting	Phase 2		Interventional	hU-MSCs (stem cell educator therapy)		IV	1	N/A	20		Randomized	Controlled
NCT04625738	Not yet recruiting	Phase 2	Moderate to severe	Interventional	WJ-MSCs		IV	3	1*10e6 cells/kg, 0.5*10e6 and 0.5*10e6	30	France	Randomized	Controlled
NCT04492501	Completed	Not applicable	Moderate and severe	Interventional	MSCs either alone or in combination with other novel therapies		IV	1	2*106 cells/kg	600	Pakistan	Non-randomized	Controlled

Abbreviations: *AT-MSCs* adipose tissue-derived mesenchymal stem cells, *AT-MSCs-Exo*: adipose tissue mesenchymal stem cell-derived exosomes, *BM-MSCs* bone marrow-derived human mesenchymal stem, *CM* conditioned medium, *DPSCs* human dental pulp mesenchymal stem cells, *ECV* extracellular vesicle, *hP-MSCs* human placental-derived mesenchymal stem cells, *hUC-MSC* human umbilical cord-derived mesenchymal stem cell, *hESCs* human embryonic stem cells, *iPSCs* induced pluripotent stem cells, *IMRCs* immunity- and matrix-regulatory cells, *OM-MSCs* olfactory mucosa-derived mesenchymal stem cells, *WJ-MSCs* Wharton's Jelly-derived mesenchymal stem cell. Data are compiled from https://clinicaltrials.gov/ct2/results?cond=covid-19+AND+stem+cells&term=&cntry=&state=&city=&dist=

The MSC dose (3–25 × 10^6^ cells/kg) varies widely. More than 14 trials have injected 1 million cells/kg, while others have injected 0.5–3, 25–90, or 100–400 million cells/kg [63]. The number of administrated doses varies between one and five; the majority of trials have administered one to three doses: 13 trials, a single dose; 14 trials, two doses; 11 trials, three doses; 6 trials, four doses; and 3 trials, five doses [63].

The administration route in most trials is IV to minimize invasiveness, while optimizing cell retention and observable migration to the affected area [64]. A few trials have used intramuscular (IM) administration, while others have used inhalation and jet nebulization. MSCs have been sourced from the BM, umbilical cord, AD tissue, dental pulp, and pooled olfactory mucosa [63]. Many trials have used hUC-MSCs, particularly from Wharton’s jelly (WJ-MSCs) [63]; the most common MSC sources in descending order are the umbilical cord, BM, and AD tissue [63]. The vast majority of the MSCs used are allogeneic, while few are from autologous sources [63]. It is unclear which source is superior in COVID patients. The autologous source has the benefits of source availability and the absence of immune rejection and ethical controversy, although it is difficult to obtain a large number of cells, it is not helpful in emergencies, and it needs a biopsy, which exposes patients to risk. In contrast, the allogeneic source has the benefits of high cell availability, high-consistency materials, high patient throughput, no need for biopsy, and commercial availability [65]. A meta-analysis by McIntyre et al. suggests that the allogeneic source provides desirable outcomes compared with other cell sources (autologous, xenogeneic, or syngeneic). Therefore, allogeneic MSC sources might be promising in COVID-19 [66]. hUC-MSCs express the least major histocompatibility complex (MHC)-I, so using them as an allogeneic source does not cause an immune response [64, 67–69]. The umbilical cord is extremely rich in MSCs [68], is easily obtained, and is otherwise considered medical waste. It is, therefore, free of ethical concerns, unlike embryonic stem cells. Accordingly, hUC-MSCs represent a prospective source for MSCs to be exploited in cell-based therapy [64, 67, 70, 71].

Several of the trials use MSCs due to their safety, efficacy, and potential for disease amelioration, boosting the immune system via immune cell reprogramming and relieving symptoms. Their differentiation ability and regenerative capacity also contribute to disease amelioration. MSCs migrate to damaged tissues, induce tissue repair, and exhibit an antiapoptotic effect without AEs.

## MSC-derived exosomes and COVID-19

Despite the enormous success of MSCs in alleviating diseases, there are concerns regarding their safety, therapeutic efficacy, durability, and scalability [57, 58, 72]. Their therapeutic potential is primarily due to their secreted extracellular vesicles (EVs) [73]. EVs secreted from different cell sources are considered important messengers in intercellular communication as they transfer bioactive lipids, proteins, and nucleic acids. EVs include (1) exosomes, with a diameter of 40–150 nm, which are released into the extracellular environment when multivesicular bodies fuse with the cell membrane, and (2) microvesicles, with a diameter of 150–1000 nm, developing from direct budding of the plasma membrane. MSC-derived exosomes have several advantages: exosomes avoid MSCs’ AEs, are nanoparticles with the ability to penetrate the blood–brain barrier, and avoid potential pulmonary embolism related to MSC transplantation. MSC-derived exosomes contain many bioactive molecules, such as lipids, proteins, mRNAs, long-noncoding RNAs, microRNAs, and mitochondrial DNA [74, 75]. MSC-derived exosomes exert anti-inflammatory and immunomodulatory effects in preclinical studies on myocardial infarction (MI), ischemia, cancer, lung injury, etc. [76, 77]. Exosomes exceed MSCs in sustainability and scalability as they are more stable than MSCs [25, 78]. However, their tumorigenic potential is debatable, as some studies support their tumor promotion potential, whereas others support their tumor inhibitory potential [79]. Preclinical and clinical studies have demonstrated the effects of exosomes in reducing cytokine storm complications, such as alveolar inflammation, edema, and epithelial tissue regeneration in inflammatory diseases, such as ARDS, asthma, COPD, and acute lung injury (ALI) [25, 80–86]. Therefore, clinical trials may start to use MSC-derived exosomes to attenuate the cytokine storm in severe COVID-19.

There are some challenges in using exosomes. First, they modulate the immune response toward tolerance and homeostasis [87–90]. This response is desirable in non-infectious diseases, such as graft-versus-host disease, and are beneficial in infectious diseases, such as influenza [84, 91–93]. However, other viruses or bacteria might not respond in the same manner, because unconstrained replication may occur [87]. Second, MSCs are heterogeneous, and MSC-derived exosomes show heterogeneity. Variability is observed between different sources, such as the BM and AD tissue [94], or even in the same population or from the same source but different donors [95]. BM-MSC-derived EVs from different donors exhibit different cytokine contents, which affects their potency [87, 91]. AEs also can differ; for instance, AD-MSC-derived EVs show more thrombogenic markers and more significant thrombogenic potential than BM-MSC-derived EVs [96]. This AE may pose a significant risk in COVID-19 as these patients are already at risk of thrombosis [97]. Therefore, an immortalized clonal MSC-derived EV line should be created to avoid potency variations and standardize the therapy [87].

A nonrandomized prospective study assessed the safety and efficacy of a BM-MSC-derived exosomal agent (ExoFlo) in 24 moderate-to-severe and severe COVID-19 patients. The patients were injected with 15 mL of ExoFlo and monitored for 14 days post-injection. No AEs were observed in the first 72 h post-injection. The majority of patients clinically recovered with improved oxygenation. Laboratory values of absolute neutrophil count, CRP, ferritin, and d-dimer decreased, while lymphocyte counts increased. This study demonstrated the potential safety and efficacy of BM-MSC-derived exosomes, which may be a promising therapeutic approach for COVID-19 [86].

However, the International Society for Cellular and Gene Therapies (ISCT) and the International Society for Extracellular Vesicles (ISEV) highlighted some issues with ExoFlo, such as insufficient data about Food and Drug Administration (FDA) approval, biological characteristics compared with other products, characterization, cell source evidence, and accurate dose (concentration/mL), and with the study, such as missing electrocardiogram (ECG) and pulse oximetry data. In addition, the ISCT and the ISEV questioned how events that occurred more than 72 h post-injection could certainly be unrelated to the exosomal agent [98]. Sengupta et al. [86] reported that ExoFlo is prepared by FDA-approved manufacturing facilities that meet current good manufacturing practice (cGMP) guidelines. They also provided light scatter and fluorescence data to confirm ExoFlo’s characterization. Proteomic analysis revealed the presence of proteins with immunoregulatory, cell migration, angiogenesis, cell differentiation, and apoptosis regulation functions. In addition, the dose of ExoFlo was 15 mL, with a concentration of ~ 40 million cells/mL. The authors also confirmed that the patients’ vital signs, oxygen saturation, and ECG were regularly monitored post-injection for 14 days [99].

## Future approaches to enhancing MSC potency for COVID-19 treatment

### MSC coating

Despite the ability of MSCs to migrate to injury sites, the amount of engraftment is relatively low. For instance, only 1% of infused MSCs were found at the MI site in rats 4 h after infusion [100]. For MSCs to migrate to injured tissue, the tissue must first chemoattract MSCs from the blood circulation, which depends on several MSC surface markers, including L-selectin, CD44, CD24, CD49a–f, CD29, CD18, intercellular adhesion molecule (ICAM)-1, ICAM-2, and vascular cell adhesion molecule (VCAM)-1, and their interaction with specific target tissue markers [101].

Studies on cell surface modification of MSCs have shown promise in preclinical models [102]. Coating MSCs with sialyl Lewis X (SLeX), an essential mediator found on the leukocyte cell surface involved in leukocyte migration to inflamed tissues, is the first step in the migration process. Biotin-avidin technology can also be used to supply hMSCs with biotinylated lipid vesicles to facilitate linking to streptavidin–SLeX and increase migration toward the P-selectin substrate [103]. Similarly, in vivo, SLeX-engineered MSCs demonstrate enhanced migration to the inflamed endothelium compared with naive MSCs [104].

Another method of MSC coating is through antibodies targeted to antigens expressed in target sites. An in vitro study evaluated the migration of ICAM-1–MSCs to human umbilical vein endothelial cells (HUVECs) and detected enhanced binding. The binding intensified when HUVECs were pretreated with TNF-α to stimulate ICAM expression (105). An in vivo study used anti-VCAM-1 to coat MSCs before infusion in an experimental colitis and inflammatory bowel disease model. Increased migration of anti-VCAM-1-coated MSCs to injury sites was observed, with no AEs on MSC characteristics, morphology, or viability [106, 107]. Therefore, MSC surface modifications can be a promising strategy for enhancing their therapeutic efficacy in COVID-19.

Finally, strategies using biodegradable/biocompatible MSC coatings have proven beneficial in MSC retention in cardiac tissue and could be modified to enhance their retention in lung tissue after IV administration [98, 108].

### Genetically modified MSCs

Several studies have used genetically modified MSCs to enhance the expression of a specific therapeutic protein or deliver therapy aimed at a specific disease [109]. Genetic modification of MSCs can be performed via viral vector or nonviral delivery. In viral vector delivery, insertional mutagenesis, immunogenicity, and limited carrying capacity are the main concerns. These concerns are minimal in nonviral delivery, which includes liposomes, plasmids, and miniplasmids; however, this approach has low transfection ability and transitory transgene expression (110). Due to MSC tropism in cancer tissues, several preclinical studies have investigated the possibility of using genetically engineered MSCs to target diverse types of cancer [111–113]. In a murine xenograft melanoma model, tumor growth reduced following local transplantation of INF-β-transduced hMSCs [114]. Similarly, MSCs were engineered to express TNF-related apoptosis-inducing ligand (TRAIL), which can induce apoptosis in cancer cells but not in healthy cells, and were proven efficacious in some preclinical studies [115, 116].

Genetically modified MSCs have also been tested in other diseases, such as Alzheimer’s disease [117], neurodegenerative diseases [118], acute cardiovascular diseases [119], spinal cord injury [120], and systemic lupus erythematosus [121]. Lotfy et al. [36] reported that the immunomodulatory and neuroprotective effects of genetically modified MSCs can could be enhanced in vivo by genetically modulating important inflammatory targets, such as INF-β and sphingosine kinase-1 (SPK1) [122, 123].

Several studies have assessed genetically modified MSCs in ARDS and radiation-induced lung injury. ACE2 has protective activity against severe acute lung injury [124]. ACE2-overexpressing hUC-MSCs played a more therapeutic anti-inflammatory role than unmodified MSCs in murine lung injury models [124, 125]. KGF plays a substantial role in lung epithelial cell repair and proliferation, and angiopoietin-1 sustains endothelial maturation and permeability [50, 126]. Both KGF-MSCs and angiopeotin-1-MSCs showed enhanced pulmonary vascular permeability and modulated pulmonary inflammation. There was a significant reduction in inflammatory mediators, including Cxcl2, IL-6, IL-1β, IFNγ, and TNF-α, in angiopoietin-1-MSCs compared with native MSCs. In another study, MSCs overexpressed decorin, a natural compound that attenuates fibrosis by inhibiting collagen-1, α-smooth muscle actin (α-SMA), and TGF-β1 [127].

More preclinical studies are required to implement genetic modification of MSCs to make them more potent therapeutic agents for COVID-19 treatment.

### MSCs and nanotechnology

Nanotechnology can be exploited to improve the therapeutic efficacy and enhance the delivery of MSCs in COVID-19 in order to augment their therapeutic effects, ameliorate symptoms, and decrease mortality. Since the cytokine storm represents a significant risk for COVID-19 patients, there is an urgent need to inhibit it. Metcalfe et al. suggested that nanosynthetic stem cells (LIFNano) could inhibit and modulate the cytokine storm seen in COVID-19 [72]. LIFNano carries leukemia inhibitory factor (LIF) with 1000 times more potency than the soluble LIF released from MSCs. Quinton et al. demonstrated that endogenous LIF plays a crucial role in lung protection in acute lung injury [128]. LIFNano showed a therapeutic effect in a multiple sclerosis experimental animal model [129]. Therefore, LIFNano represents an alternative to MSC therapy because of its high volume and ability to inhibit the cytokine storm and repair damaged lung tissue [72].

Valizadeh et al. revealed that the nanocurcumin ameliorates the cytokine storm and decreases the expression and secretion of IL-6 and IL-1β but not IL-18 and TNF-α in both serum and supernatant [130].

Nanocarriers have a wide range of applications and represent a delivery platform for drugs, vaccines, and cells because of their sustained release, selectivity, and specificity. Chitosan plays a substantial role in drug delivery into lung tissue in infectious diseases as it is a biodegradable, biocompatible, and safe polymer. It acts as a pulmonary particulate carrier for drugs because of its mucoadhesive effect and its ability to locate into the specific site, as well as its permeation [131]. Therefore, combining MSCs with chitosan hydrogel could enhance their therapeutic efficacy, permeation, adhesion, and targeting. Mehta et al. predicted that polysaccharide nanoparticles, nanotheranostics, and mesoporous silica nanoparticles would be promising targeted nanocarriers and drug delivery systems in COVID-19. Therefore, their combination with MSCs might pave the way for a new COVID-19 treatment [132]. Some nanomaterials display antiviral efficacy, such as gold nanoparticles and heparan sulfate proteoglycan (HSPG) [133]. Therefore, combining MSCs with nanomaterials that exhibit antiviral activity might duplicate and enhance their therapeutic efficacy.

### MSC preconditioning

MSCs can also be preconditioned with other compounds to synergize their effect or enhance the overall outcome for COVID-19 patients. For instance, MSCs can be preconditioned with vitamin D, which acts as a strong immunomodulator [134]. Since MSCs might undergo apoptosis after transplantation, pretreatment with antioxidants might help protect them. Mohammadi et al. demonstrated that astaxanthin (ATX), a potent antioxidant, plays a protective and supportive role for AD-MSCs by overcoming oxidative stress; decreasing hydrogen peroxide, which induces cell apoptosis; and enhancing the expression of native cell antioxidants, such as heme oxygenase-1 (HO-1) and reduced nicotinamide adenine dinucleotide phosphate (NADPH) quinine oxidoreductase 1 (NQO1). Similar approaches could help protect MSCs and enhance their viability in harsh conditions and, hence, improve their therapeutic efficacy in COVID-19 [135]. Interestingly, selenium is considered an antioxidant with a low toxicity profile and antiviral property. Therefore, selenium could be used, either in regular form or preferably in its nanoform as nanoselenium (nanoSe) [136], in combination with MSCs to ameliorate COVID-19 symptoms.

## Conclusion

SARS-CoV-2 emerged in Wuhan, China, and has become a life-threatening virus, causing the COVID-19 pandemic with significant morbidity and fatality rates. MSCs are the most commonly used stem cells in clinical trials, with validated safety, can migrate to sites of tissue injury, and can ameliorate the COVID-19-associated cytokine storm via their paracrine immunomodulatory effect. In fact, multiple studies are underway exploring the therapeutic efficacy of MSCs in patients with moderate-to-severe COVID-19. A cell-free approach, such as using MSC-derived exosomes, promises similar therapeutic efficacy with fewer AEs. Future studies designed to enhance MSC therapy in COVID-19 can take advantage of advances in nanotechnology and cell surface and genetic modifications of MSCs to enhance their retention, survival, and immunomodulatory effects and to further improve their therapeutic efficacy in COVID-19.

## Supplementary Information

**Additional file 1.** Properties of published studies of MSCs for COVID-19; including patient characteristics, MSCs characterization, and explanation of adverse events and serious adverse events.

**Additional file 2.** Detailed Excel sheet of the ongoing clinical trials with MSCs interventions in COVID-19 patients.

## Data Availability

All data presented in this review are totally available and present in the text.

## Abbreviations

COVID-19: Coronavirus disease 2019
SARS-CoV-2: Severe acute respiratory syndrome coronavirus-2
MSC: Mesenchymal stem cell
ICU: Intensive care unit
TNF-α: Tumor necrosis factor alpha
IL: Interleukin
MIP: Macrophage inflammatory protein
MCP1: Monocyte chemoattractant protein 1
COPD: Chronic obstructive pulmonary disease
ARDS: Acute respiratory distress syndrome
mRNA: Messenger RNA
G-CSF: Granulocyte colony-stimulating factor
GM-CSF: Granulocyte–macrophage colony-stimulating factor
IL-1RA: Interleukin 1 receptor type 1
FGF-2: Fibroblast growth factor 2
VEGF-A: Vascular endothelial growth factor A
IFNγ: Interferon gamma
IP10: IFNγ-induced protein 10
PDGFB: Platelet-derived growth factor B
HLA: Human leukocyte antigen
IBD: Inflammatory bowel disease
T1D: Type 1 diabetes
PGE2: Prostaglandin E2
IDO: Indolamine 2,3-dioxygenase
hUC-MSC: Human umbilical cord tissue-derived MSC
BM: Bone marrow
KGF: Keratinocyte growth factor
MMP-9: Matrix metalloproteinase-9
HGF: Hepatocyte growth factor
PaO_2_: Arterial partial pressure of oxygen
FiO_2_: Fraction of O_2_ inspiration
RR: Respiratory rate
CT: Computed tomography
CRP: C-reactive protein
IV: Intravenous
AST: Aspartate transaminase
ALT: Alanine transaminase
EV: Extracellular vesicle
MHC: Major histocompatibility complex
ALI: Acute lung injury
ISCT: International society for cellular and gene therapies
ISEV: International society for extracellular vesicles
FDA: Food and Drug Administration
cGMP: Current good manufacturing practice
ECG: Electrocardiogram
MI: Myocardial infarction
ICAM: Intercellular adhesion molecule
VCAM: Vascular cell adhesion molecule
SLeX: Sialyl Lewis X
HUVEC: Human umbilical vein endothelial cell
SPK1: Sphingosine kinase-1
TGF-β: Transforming growth factor beta
α-SMA: α-Smooth muscle actin
LIF: Leukemia inhibitory factor
HSPG: Heparan sulfate proteoglycan
HO-1: Heme oxygenase-1
NADPH: Reduced nicotinamide adenine dinucleotide phosphate
NQO1: NADPH quinine oxidoreductase 1
AT-MSCs: Adipose tissue-derived mesenchymal stem cells
AT-MSCs-Exo: Adipose tissue mesenchymal stem cell-derived exosomes
BM-MSCs: Bone marrow-derived human mesenchymal stem
CM: Conditioned medium
DPSCs: Human dental pulp mesenchymal stem cells
ECV: Extracellular vesicle
hP-MSCs: Human placental-derived mesenchymal stem cells
hUC-MSC: Human umbilical cord-derived mesenchymal stem cell
hESCs: Human embryonic stem cells
iPSCs: Induced pluripotent stem cells
IMRCs: Immunity- and matrix-regulatory cells
OM-MSCs: Olfactory mucosa-derived mesenchymal stem cells
WJ-MSCs: Wharton's Jelly-derived mesenchymal stem cell
