# Supplementary material for: Mesenchymal stem cell-based therapy and exosomes in COVID-19: current trends and prospects
Source: Stem Cell Res Ther. 2021 Aug 21;12:469. doi: 10.1186/s13287-021-02542-z (PMC8379570; doi:10.1186/s13287-021-02542-z)
Supplement: Supplementary file 1 — Additional file 1. Properties of published studies of MSCs for COVID-19; including patient characteristics, MSCs characterization, and explanation of adverse events and serious adverse events. [file 13287_2021_2542_MOESM1_ESM.docx]

**Table 1: Baseline and patient characteristics for published MSC & COVID-19 studies**

| **Follow up (after MSCs' infusion)** | **Baseline general** | **Baseline pulmonary function (treatment), (control)** | **Comorbidities** | **Age median (years) (treatment), (control)** | | **Study type** | **Country** | **Study** |
| --- | --- | --- | --- | --- | --- | --- | --- | --- |
| 17 days (from day 13 to day 30). | Fever (temperature: 38.2^°^C),  SOB, cough, diarrhea | SPO_2_: 81% | Moderate anemia, hypertension and  Diabetes. | 65 t and c | | Case report | China | [1] |
| 14 days | Fever:  **treatment:** temperature: (37.7-39),  **Control:** temperature: (36-38.9)], Cough and SOB | SPO_2_: (89-95%), (91-93%) | Hypertension (for the critically ill patient), NR for other patients. | 55 t, 60.5 c | | Pilot trial. | China | [2] |
| 14 days | Fever:  [10 of treatment group and 26 of control group; temperature ≥ 37.3 °C], Cough and SOB | SPO_2_≤ 93%  (11 of treatment group and 27 of the control group required oxygenation). | Hypertension and diabetes. | 65 t, 58 c | | Pilot trial. | China | [3] |
| 31 days | NR | PaO_2_/FiO_2_ in treated group 124 (68-164)  PaO2/FiO2 in control: 108.5 (68.5-165.5 | Diabetes, hypertension, obesity, cancer, heart disease | 59.5 t, 58 c | | A double blind, phase 1/2a  randomized controlled trial | The United States | [4] |
| NR | NR | NR | Diabetes, hypertension, fatty liver disease, asthma | | 42 t, 50 c | Phase 1 clinical trial | China | [5] |
| 28 days | NR | Mean oxygenation index : 258 | Diabetes, hypertension, chronic kidney disease, hepatitis B, bronchial asthma, alzheimer's disease, anemia. | | 61.7 (mean) | Pilot trial | China | [6] |

**Abbreviation:**
T: treatment group, C: Control group, SPO_2_: Oxygen saturation, SOB: Shortness of breath, NR: Not reported, PaO2/FiO2: Ratio of arterial oxygen partial pressure to fractional inspired oxygen

**Table 2: Product (MSCs) characterization for published MSC & COVID-19 studies**

| **Viability** | **Control** | **Administration** | **Characterization** | **Culture media** | **Passage (P)** | **Donor gender** | **MSCs' dose and frequency** | **MSCs'source** | **Study** |
| --- | --- | --- | --- | --- | --- | --- | --- | --- | --- |
| More than 90%. | None | IV | **More than 95%** **were positive for** CD90, CD105, CD44, CD73.  **Less than 2% of them were positive** for CD45, CD19, CD34, CD11b, and HLA-DR. | Minimum essential medium-α and 5% human platelet lysate | P 5 | Female | 5 × 10^7^ cells each time – every three days (day 13-16-19). | hUMSCs | [1] |
| NR | Controlled | IV | NR | NR | NR | NR | 1 × 10^6^ cells/Kg- Once | NR | [2] |
| NR | Controlled | IV | **More than 95% were positive for:** CD73, CD90, and CD105.  **Less than 2% of them were positive:**  for CD34, CD45, CD14 or CD11b, CD79α or CD19, and HLA-DR. | NR | P 3-5 | NR | 2 × 10^6^ cells/kg- once | hUMSCs | [3] |
| More than 80% | Controlled | IV | Cell surface markers:  CD90/CD105 > 95%  CD34/CD45 < 5%  Endotoxin (<1.65 EU/mL)  Gram stain: negative | NR | NR | NR | 100 ± 20 × 10^6^ cells  2 doses (day 0 and 3) | UC-MSCs | [4] |
| NR | controlled | IV | Fibroblast-like morphology  Cell surface markers: CD19, CD34, CD11b, CD45, CD73, CD105, CD90, and HLA-DR | MSCs culture  medium | P5 | NR | 3 × 10^7^ cells per dose  3 doses  (0, 3, and 6) days. | UC-MSCs | [5] |
| NR | None | IV | NR | NR | NR | NR | 1 × 108 cells  4 doses (one day interval in between). | UC-MSCs | [6] |

**Abbreviation:**hUMSCs: Human umbilical cord mesenchymal stem cells, IV: intravenous, NR: Not reported, UC-MSCs: Umbilical cord mesenchymal stem cells

**Table 3: Explanation of adverse events and serious adverse events for published MSC & COVID-19 studies.**

| **Serious adverse events (SAEs) treatment related** | **Adverse events (AEs) non-treatment related** | **Adverse events (AEs) treatment related** | **Study** |
| --- | --- | --- | --- |
| None | Immunosuppression, acute gastrointestinal bleeding and severe organ injury. | None | [1] |
| None | None | None | [2] |
| None | None | None | [3] |
| 2 SAEs observed in 2/12 UC-MSCs- treated patients.  16 SAEs in 8/12 patients in the control group | 53 AEs in 11 patients including an increase in  vasopressor dose, new cardiac  arrhythmia requiring  cardioversion, new ventricular  tachycardia, ventricular  fibrillation, or asystole | 35 AEs in 8 patients such as an increase in  vasopressor dose | [4] |
| None | Mechanical ventilation: 4 patients  Fever: 2 patients  Fatigue: 5 patients  Cough: 8 patients  Shortness of breath: 5 patients | Transient facial flushing: 2 patients  Transient hypoxia in 1 patient.  Mechanical ventilation: 1  Fever: 5 patients  Fatigue: 4 patients  Cough: 4 patients  Shortness of breath: 1 patient | [5] |
| None | Bacterial pneumonia and septic shock led to death. | Hypoproteinemia, sleeplessness, gastrointestinal disease and paroxysmal arrhythmia.  (it is not clear whether they are treatment-related or not) | [6] |

**Abbreviation**AEs: Adverse events, SAEs: Serious adverse events, UC-MSC: umbilical cord mesenchymal stem cells,

**References**

[1] B. Liang *et al.*, “Clinical remission of a critically ill COVID-19 patient treated by human umbilical cord mesenchymal stem cells: A case report,” *Medicine (Baltimore).*, vol. 99, no. 31, p. e21429, 2020, doi: 10.1097/MD.0000000000021429.

[2] Z. Leng *et al.*, “Transplantation of ACE2- Mesenchymal stem cells improves the outcome of patients with covid-19 pneumonia,” *Aging Dis.*, vol. 11, no. 2, pp. 216–228, 2020, doi: 10.14336/AD.2020.0228.

[3] L. Shu *et al.*, “Treatment of severe COVID-19 with human umbilical cord mesenchymal stem cells,” *Stem Cell Res. Ther.*, vol. 11, no. 1, pp. 1–11, 2020, doi: 10.1186/s13287-020-01875-5.

[4] G. Lanzoni *et al.*, “Umbilical cord mesenchymal stem cells for COVID-19 acute respiratory distress syndrome: A double-blind, phase 1/2a, randomized controlled trial,” *Stem Cells Transl. Med.*, vol. 10, no. 5, pp. 660–673, 2021, doi: 10.1002/sctm.20-0472.

[5] F. Meng *et al.*, “Human umbilical cord-derived mesenchymal stem cell therapy in patients with COVID-19: a phase 1 clinical trial,” *Signal Transduct. Target. Ther.*, vol. 5, no. 1, 2020, doi: 10.1038/s41392-020-00286-5.

[6] Y. Feng *et al.*, “Safety and feasibility of umbilical cord mesenchymal stem cells in patients with COVID-19 pneumonia: A pilot study,” *Cell Prolif.*, vol. 53, no. 12, pp. 1–8, 2020, doi: 10.1111/cpr.12947.
